# Co-relation of Monocyte Count in High vs. Low Thrombus Burden ST-Segment Elevated Myocardial Infarction (STEMI) Patients Undergoing Primary Percutaneous Coronary Intervention

**DOI:** 10.7759/cureus.24344

**Published:** 2022-04-21

**Authors:** Muhammad Zeeshan, Sara Yousaf, Adeel Ahmed, Hina Bahadar, Usman Ali, Sidra Jabeen, Hassan ul Hussain, Hassan Mumtaz, Mohammad Hasan

**Affiliations:** 1 Cardiology, KRL Hospital, Islamabad, PAK; 2 Internal Medicine, Shifa International Hospital, Islamabad, PAK; 3 Internal Medicine, DHQ (District Headquarters) Teaching Hospital, Gujranwala, PAK; 4 Medical Unit, Mayo Hospital, Lahore, PAK; 5 Internal Medicine, Bahria International Hospital, Rawalpindi, PAK; 6 Emergency Medicine, Liaquat National Hospital and Medical College, Karachi, PAK; 7 Medicine, Dow University of Health Sciences, Civil Hospital Karachi, Karachi, PAK; 8 Urology, Guys & St Thomas Hospital, London, GBR; 9 General Practice, Surrey Docks Health Center, London, GBR; 10 Public Health, Health Services Academy, Islamabad, PAK; 11 Clinical Research Center, Maroof International Hospital, Islamabad, PAK; 12 Internal Medicine, Jinnah Postgraduate Medical Centre, Karachi, PAK; 13 Internal Medicine, Jinnah Sindh Medical University, Karachi, PAK

**Keywords:** interventional cardiologist, diagnostic imaging, coronary thrombosis, percutaneous coronary intervention, monocytes, myocardial infarction

## Abstract

Introduction

Primary percutaneous coronary intervention (PPCI) in ST-elevation myocardial infarction (STEMI) patients can lead to poor outcomes. Intra-coronary thrombus development due to atherosclerotic plaque rupture and coronary blood flow blockage causes STEMI. Intracranial thrombosis in STEMI patients is fatal.

It was our goal to establish how often patients with STEMI underwent PPCI with a high thrombus burden versus a low thrombus burden and to compare the mean monocyte count between the two groups.

Material and methods

This cross-sectional study was conducted at KRL Hospital Islamabad from October 2021 to March 2022. At a 95% level of confidence, a 5% margin of error, and keeping a population size of 330, a sample size of 178 was obtained using the Raosoft sample size calculator (Raosoft, Inc., Seattle, WA). The non-probability consecutive sampling method was used.

All patients with STEMI undergoing PPCI, aged between 18 and 80 years, and presenting within 24 hours of symptoms were included in our study. Pre-PPCI pharmacological treatment given within three hours of the onset of a heart attack to stabilize patients with myocardial infarction included aspirin, clopidogrel, and an intravenous bolus of 70 U/kg of body weight of un-fractionated heparin. The collected data were analyzed using SPSS version 26.0 (IBM Corp., Armonk, NY). Fisher's exact test was employed, and a p-value of less than 0.05 was deemed statistically significant. The odds ratio and confidence interval were also calculated.

Results

A total of 178 participants were included in the research, out of which males were predominant with more than half of the study population. The mean age in patients having a low thrombus burden was 37.75 ± 6.39 years and that of patients with a high thrombus burden was mean 56.04 ± 7.98 years. In high thrombus burden patients, diabetes mellitus was found in 98.3%, hypertension in 120 patients (100%), obesity in (60%), and tobacco consumption in 120 patients (100%). The mean monocyte count in high burden patients was 70.27 ± 3.24, whereas it was 61.89 ± 5.71 in low burden patients. Only five patients had a Thrombolysis In Myocardial Infarction (TIMI) score of 5 while 34.8% of patients arrived in three to six hours and 12.9% arrived in less than three hours. Patients with a high monocyte count have 1.3 times more chances of developing the disease when the monocyte count was high (OR = 1.318, 95% CI = 1.140-1.524).

Conclusion

Patients with STEMI undergoing PPCI had a higher monocyte count upon admission, which was an independent clinical predictor of a high thrombus burden. Our findings suggest that admission monocyte count may be available for early risk stratification of high-thrombus burden in acute STEMI patients and might allow the optimization of anti-thrombotic therapy to improve the outcomes of PPCI.

## Introduction

As atherosclerotic plaques rupture and coronary blood flow are disrupted, an intra-coronary thrombus is formed, which is the fundamental etiology of ST-elevated myocardial infarction (STEMI) [1-2]. Patients with STEM who have a large volume of intra-coronary thrombus have a worse prognosis [3]. Many pharmacological and surgical treatments, such as glycoprotein IIB/IIIA antagonists and thrombectomy, have been developed for intracoronary thrombus management. STEMI management may be helped by predicting the intra-coronary thrombus burden. As recently established [4], the thrombus load might be predicted using red cell distribution width (RDW) and bilirubin levels as recently established [4-5].

Myocardial infarction has been associated with a greater count of monocytes [6] and the pathophysiology of coronary artery disease [7]. An important role has been established in both the synthesis of procoagulant substances (such as tissue factors) and the stimulation of inflammation in thrombotic diseases. Monocyte counts on admission have been shown to independently predict the absence of reflow following primary PPCI [8]. Monocytes contribute significantly to immunity through the secretion of pro-inflammatory and pro-oxidant cytokines. An atherosclerotic plaque has a large number of these cells. Patients with monocytosis have been demonstrated to have a greater monocyte count, which has been linked to the formation of plaque during the acute phase of myocardial infarction (AMI) [9-10].

Researchers found that 34.8% of patients who received primary percutaneous coronary intervention for a heart attack had a significant thrombus burden. On admission, the mean monocyte count differed significantly across patients with high and low thrombus burdens (0.61-0.29 and 0.53-0.24, respectively) [11]. Monocyte counts were higher in patients with high levels of fibrinolysis compared to lower levels in individuals with low levels of fibrinolysis [12].

Those with a high thrombus burden had a greater monocyte count compared to patients with a low thrombus burden, according to many studies. Monocyte count may have a deleterious effect on patients with a high thrombus burden. Literature, on the other hand, hasn't yielded much evidence. It's also worth noting that there are no local data in this area that could assist us in determining whether or not an abnormal monocyte count has any negative impact on health. The mean monocyte count of patients with STEMI was compared between two groups, one with a significant thrombus burden and the other without.

In order to incorporate the findings in a local context, we conducted this study. Consequently, we will be able to better serve our patients and keep our prevention and treatment regimens up-to-date.

## Materials and methods

From October 2021 to March 2022, KRL Hospital, Islamabad, conducted cross-sectional research. At a 95% level of confidence, a 5% margin of error, and keeping a population size of 330, a sample size of 178 was obtained using the Raosoft sample size calculator (Raosoft, Inc., Seattle, WA). The non-probability consecutive sampling method was used.

Inclusion and exclusion criteria

All patients with STEMI undergoing PPCI, aged between 18 and 80 years old, and presenting within 24 hours of symptoms were included in our study.

Patients who previously had a history of myocardial infarction, recurrent infarctions, unstable or chronic stable angina, or previous objective evidence of coronary artery disease, such as coronary angiograms or stress exercise tolerance tests, nuclear stress tests, or stress echocardiography, were excluded from our study.

Data collection

A total of 178 consecutive patients from the department of cardiology who met inclusion and exclusion criteria were eligible for participation. It was necessary to secure the patients' explicit written consent for this investigation. Pre-PPCI pharmacological treatment given within three hours of the onset of a heart attack to stabilize the patients with myocardial infarction included aspirin, clopidogrel, and an intravenous bolus of 70 U/kg of body weight of un-fractionated heparin.

Primary procedure

As is customary, an 8-French guiding catheter was inserted via the conventional radial access. All patients received a stent as part of their treatment. There was an intracoronary thrombus load evaluation for all patients who had ante-grade flow via guidewire or small-balloon dilation. Before receiving aspirin and clopidogrel, all patients in the emergency department had their monocyte count taken in standard ethylenediaminetetraacetic acid (EDTA)-containing tubes.

Data analysis

In SPSS version 26.0 (IBM Corp., Armonk, NY), the acquired data were input and evaluated. In order to show numerical data, such as age and monocyte count, the mean and standard deviation (S.D) were utilized. Qualitative variables, such as diabetes mellitus, hypertension, gender, obesity, smoking, and thrombus burden, will be presented as percentages and frequencies, respectively. An independent T-test was applied to see the significance, defined as an effect size of 0.05 or more. After stratification, Fisher's exact test was employed. In this experiment, a p-value of less than 0.05 was deemed statistically significant. Fischer’s exact test investigates if the odds ratio is equal to 1 or not, which tells us what are the odds of developing the disease when a specific risk factor is involved.

## Results

A total of 178 participants were included in the research, out of which males were predominant with more than half of the study population. The mean age of patients having low thrombus burden was 37.75 ± 6.39 years and that of patients with a high thrombus burden was 56.04 ± 7.98 years. The independent T-test applied showed a significant p-value. In high thrombus burden patients, diabetes mellitus was found in 98.3%, hypertension in 120 patients (100%), obesity in (60%), and tobacco consumption in 120 patients (100%). Fisher’s test showed a significant association of high thrombus burden with hypertension, diabetes, obesity, and tobacco consumption. The mean monocyte count in high burden patients was 70.27 ± 3.24, whereas it was 61.89 ± 5.71 in low burden patients. The independent T-test applied showed a significant p-value as depicted in Table 1.

**Table 1:** Patient Demographics and Clinical Characteristics

	Low Thrombus Burden (n = 58)	High Thrombus Burden (n = 120)	P-Value
Mean Age (Years ± SD)	37.75 ± 6.39	56.04 ± 7.98	0.00
Sex Male	0	97 (80.83%)	0.00
Female	58 (100%)	23 (19.17%)	
Diabetes Mellitus Yes	0	118 (98.33%)	0.00
No	58 (100%)	2 (1.67%)	
Hypertension. Yes	5 (8.63%)	120 (100%)	0.00
No	53 (91.37%)	0	
Obesity Yes	0	72 (60%)	0.00
No	58 (100%)	48 (40%)	
Tobacco Yes	12 (20.69%)	120 (100%)	0.00
No	46 (79.31%)	0	
Mean Monocyte Count	61.89 ± 5.71	70.27 ± 3.24	0.00

Sixty-two patients had a TIMI score of 3 (34.8%), 53 patients (29.8%) had a TIMI score of 4, whereas only five patients (2.8%) had a TIMI score of 5, as listed in Table 2.

**Table 2:** TIMI Thrombus Scale TIMI: Thrombolysis In Myocardial Infarction

TIMI Score	Frequency	Percent
1	6	3.4
2	52	29.2
3	62	34.8
4	53	29.8
5	5	2.8

One-hundred-one patients (56.7%) presented to the hospital within six to 12 hours, whereas 54 (30.3%) patients presented in three to six hours and only 23 (12.9%) patients presented to the hospital in less than three hours, as presented in Table 3.

**Table 3:** Time From Symptom Onset to PPCI PPCI: Primary Percutaneous Coronary Intervention

Time from Onset	Frequency	Percent
<3h	23	12.9
3-6h	54	30.3
6-12h	101	56.7

Patients with high monocyte count have 1.3 times more chances of developing the disease when the monocyte count was high (OR = 1.318, 95% CI = 1.140-1.524). Patients with diabetes and obesity have 0.17 and 0.4 times increased chances of developing the disease, respectively (OR = 0.17, 95% CI = 0.004-0.066; OR = 0.40, 95% CI = 0.321-0.498), whereas hypertensive patients were 11 times more prone to develop the disease (OR = 11.60, 95% CI = 5.018-26.813), as presented in Table 4.

**Table 4:** Odds Ratio and Confidence Interval of Different Disease Factors

Variables	Odds Ratio	95% Confidence Interval	P-Value
Monocyte Count	1.318	1.140-1.524	0.00
Gender	0.192	0.133-0.277	0.00
Diabetes	0.17	0.004-0.066	0.00
Obesity	0.400	0.321-0.498	0.00
Hypertension	11.600	5.018-26.813	0.00

## Discussion

A total thrombotic blockage of a coronary artery is the hallmark of acute STEMI. The purpose of PPCI in STEMI is to restore tissue perfusion promptly, save as much cardiac muscle as possible, and improve patient outcomes. Intracoronary thrombi have been linked to poor outcomes in patients who had PPCI of the underlying lesion [13-14]. During PPCI for STEMI, however, managing the thrombotic load remains difficult. People who are at high risk of developing a high thrombus burden need to be identified as soon as possible by risk assessment. Patients with STEMI who had PPCI in the presence of a large thrombus burden (infarct-related artery or IRA) were shown to have higher admission TIMI values. A high monocyte count was shown to be a significant independent predictor of angiographic high-thrombus load in a multivariate regression study. Other than Wang and colleagues [15], no other research has examined the connection between admission monocyte count and angiographic intracoronary thrombus load in PPCI. The high thrombus load group had a higher monocyte count in that research as well.

The pathophysiology of plaque lysis and consequent thrombus development has been linked to oxidative stress and inflammation [16-17]. Monocytes make up about 10% of normal blood leukocytes and are principal participants in the systemic inflammatory response. In STEMI patients, they're linked to an inflammatory reaction at the susceptible plaque [18]. Extrinsic coagulation cascade components, such as tissue factor (TF), play a critical role in the development of thrombosis in the arteries. According to a new study, monocytes are the primary source of blood TF [19]. Neutrophils in STEMI patients' coronary arteries were stained histologically by Palmerini et al [20] using tissue-factor, who discovered that while monocytes were consistently stained robustly, neutrophils were stained less consistently. Monocyte-platelet aggregates are another potential explanation for the link between monocytes and thrombus formation (MPA). Predictors of no-reflow in STEMI patients with primary PCI have been shown to be important [21]. In acute coronary syndrome (ACS) patients, monocyte-platelet aggregates (MPA) are an excellent indicator of platelet activation [22].

When monocytes are involved in the coagulation process, more than one mechanism is at play. According to Aleman et al., monocyte-derived MPs are associated with increased prothrombinase activity and faster fibrin formation [23]. Furthermore, monocytes may promote inflammatory processes, which might lead to thrombus formation. According to Mach et al. [23], human monocyte activation promoted the production of stromelysin and interstitial collagenase, which were linked to plaque destabilization and thrombotic incidents, according to Rittersma et al. [24]. Postmortem and histological examinations of thrombus specimens from STEMI patients [25] have shown that high numbers of circulating monocytes may begin days or even weeks before the onset of symptoms [15] based on these findings.

Patients with acute coronary syndrome need to have their risk factors properly assessed before receiving therapy. However well-defined the early treatment options are for patients with ST-elevation acute myocardial infarction (STEMI), stratification of risk still affects therapeutic decisions, both in the early and late stages. It is critical to accurately categorize STEMI patients in order to guide choices about transfer to a tertiary center, the length of hospitalization, and the use of interventional and pharmacological therapy. In our research, all of the patients had primary percutaneous coronary intervention as soon as feasible. Because of this, risk categorization was very helpful for these patients as they neared the end of their hospitalization.

Furthermore, the classification of patients based on their individual risk factors is critical in the planning of any future therapeutic studies. The TIMI risk score for STEMI patients was validated using data from the TIMI 9 study and data from the Intravenous nPA for Treatment of Infarcting Myocardium II (In TIME II) trial. National Registry of Myocardial Infarction-3 (NRMI-3) patients who had thrombolytic treatment for a STEMI have a strong predictive value for the TIMI risk score (C statistic 0.79) [26]. In order to predict the outcome of a STEMI after PPCI, we used the TIMI risk ratings.

Strengths and limitations

This study is one of the very first studies of its kind to observe the thrombus burden using monocyte count as predictors. Our study has a few limitations, one of which is the small sample size. Another limitation of our study is the lack of follow-up of the patients to observe cardiovascular mortality and monocyte count after PCCI. There was a lack of access to the study population's anti-thrombotic therapy history, which might have affected the intra-coronary condition of the patients during PPCI and so was not included in the risk factor analysis. Large trials could be conducted to stratify the outcomes and to make further guidelines. It's possible that larger prospective cohort research with extensive information on past anti-thrombotic medication might be more informative.

## Conclusions

On admission, the increased monocyte count of patients who had PPCI was an independent clinical predictor of an elevated thrombus load in those who had suffered STEMI. Patients with acute STEMI who have substantial thrombus load may benefit from using admission monocyte count for early risk classification and the optimization of anti-thrombotic medication to enhance PPCI outcomes. This research recommends that we include the monocyte count as a predictor of thrombus load in our standard recommendations for patients having primary percutaneous coronary intervention.
